# Thymol in Different Parts of the Rabbit Intestinal Tract: Its Effect on Intestinal Microbiota, Morphometry and Immunoglobulin A

**DOI:** 10.3390/ani16203176

**Published:** 2026-10-10

**Authors:** Radoslava Kristofova, Karin Zitterl-Eglseer, Markus Wildner, Andreas Zitterl, Monika Pogany Simonova, Andrea Laukova, Lubica Chrastinova, Rudolf Zitnan, Margareta Takacsova, Kristina Bacova, Iveta Placha

**Affiliations:** 1 Centre of Biosciences of the Slovak Academy of Sciences, Institute of Animal Physiology, Soltesovej 4–6, 040 01 Kosice, Slovakia; kristofova@saske.sk (R.K.); simonova@saske.sk (M.P.S.); laukova@saske.sk (A.L.); takacsova@saske.sk (M.T.); bacovak@saske.sk (K.B.); 2 Unit for Nutritional Physiology and Functional Plant Compounds, Centre for Animal Nutrition and Welfare, Clinical Department for Farm Animals and Food System Transformation, University of Veterinary Medicine, Veterinaerplatz 1, 1210 Vienna, Austria; karin.zitterl@vetmeduni.ac.at (K.Z.-E.); markus.wildner@gmx.at (M.W.); 3 3rd Medical Department with Metabolic Disorders and Nephrology, Karl-Landsteiner-Institute for Metabolic Diseases and Nephrology, Clinic Hietzing, Wolkersbergenstraße 1, 1130 Vienna, Austria; andreas.zitterl@gesundheitsverbund.at; 4 National Agricultural and Food Centre, Hlohovecka 2, 951 41 Nitra-Luzianky, Slovakia; rudolf.zitnan@nppc.sk

**Keywords:** thymol, absorption, intestinal tract, gut health, rabbit

## Abstract

The positive effect of thymol on gut health largely depends on its absorption from the intestinal tract and its biotransformation processes in the intestinal wall. A total of 32 rabbits were randomly divided at 35 days of age into a control (CG) and an experimental group (EG) that received thymol at 250 mg/kg feed. Thymol concentration was significantly higher in the caecum than in the ileum (*p* < 0.05), which is likely associated with caecotrophy and reduced enzymatic activity in the distal segments of the small intestine. Coliform bacteria (*p* = 0.0095) and lactic acid bacteria (LAB; *p* = 0.0002), *Staphylococcus* spp. (*p* = 0.0002), and *Enterococcus* spp. (*p* = 0.0002) in the duodenum; LAB (*p* = 0.0003) and *Enterococcus* spp. (*p* = 0.0002) in the ileum; and other *Enterobacteriaceae* (*p* = 0.0002) and LAB (*p* = 0.0068) in the caecum were significantly higher in the EG. Despite the significantly lower thymol concentration in the ileum compared to the caecum, the beneficial LAB was positively affected. A decrease in immunoglobulin A in all parts of the intestinal tract except the colon was a result of the modulation of mucosal immunity. Thymol at this concentration modulated the gut ecosystem without disrupting its beneficial components, thereby helping maintain intestinal homeostasis.

## Simple Summary

Maintaining a healthy gut is essential for animal health, welfare, and performance. Thymol, a natural compound found in many medicinal plants, is a potential alternative to antibiotic growth promoters. The results of our experimental study provided a comprehensive insight into the relationship between the biotransformation processes of thymol occurring in different segments of the rabbit intestinal tract and the composition of the gut microbiota, immunoglobulin A secretion in the intestinal mucosa, and the morphological structure of the intestinal wall. Further understanding of these interactions is essential for understanding the role of thymol in promoting a healthy intestinal environment. We found that thymol concentrations in the intestinal tract contributed to maintaining intestinal homeostasis, thereby improving the functional integrity of the intestinal barrier and modulating the mucosal immune response.

## 1. Introduction

Although considerable research has been conducted in recent years on the effects of phytoadditives in rabbits, the complex interactions among their metabolism, intestinal microbiota, and immune responses remain largely unexplored.

Microbial populations in the gastrointestinal tract (GIT) significantly influence the bioavailability and metabolism of orally administered compounds by modulating their absorption and degradation and facilitating enterohepatic recirculation. Compounds that reach the distal GIT or participate in enterohepatic circulation are most likely to interact with gut microbes [1]. Beyond its metabolic functions, the gut microbiota plays a crucial role in nutrient digestion, immune regulation, and pathogen defence, and contributes to key metabolic pathways and maintains overall host health [2].

The gut mucosal immune system, particularly secretory immunoglobulin A, is crucial for maintaining microbial balance by preventing pathogen adhesion and promoting colonisation of beneficial bacteria [3]. This immune barrier works together with mucus and antimicrobial peptides to distinguish harmless from harmful antigens, ensuring gut homeostasis [4].

The structure of the intestinal mucosa provides important information about the health status of the gastrointestinal tract, commonly referred to as a “healthy gut.” Inappropriate dietary components can rapidly damage the intestinal mucosa because it is in close contact with intestinal contents, potentially leading to significant morphological changes in the intestinal wall. These changes are typically characterised by a reduction in villus height and an increase in crypt depth. Such adverse effects are often associated with toxins produced by pathogenic microorganisms [5].

In their study, Placha et al. [6] highlighted the potential protective effects of thymol metabolites, namely thymol sulphate and thymol glucuronide, against oxidative damage in enterocytes. However, the authors suggested that this remains only a hypothesis, as it has not yet been confirmed whether these metabolites are biologically active or inactive.

Various strategies have been explored to support mucosal immunity and maintain microbial balance, including the use of natural bioactive compounds, such as feed additives. Among these compounds, thymol, a monoterpenoid phenol derived from two isoprene units, has attracted considerable attention due to its antimicrobial and immunomodulatory properties [7]. Thymol disrupts microbial membranes by interacting with membrane proteins, increasing permeability and inducing cell death [8]. Its lipophilic and hydrophobic properties enable it to act against both Gram-positive and Gram-negative bacteria [9]. However, the effectiveness of thymol depends largely on its bioavailability, which is strongly influenced by the route of administration [10]. When administered orally, thymol is rapidly absorbed in the intestinal tract but is extensively metabolised into hydrophilic metabolites, which are then excreted via the liver and kidneys [11]. Although thymol has been extensively investigated in various animal species, only limited information is available on its distribution within the intestinal wall and its immunomodulatory effects.

Given its beneficial biological properties, thymol—as a natural feed additive—is a promising candidate for modulating the gut environment in rabbits. In the present study, we focused on its bioavailability across different intestinal segments. We expect its beneficial impact on the selected intestinal bacteria species, morphometric parameters in the jejunal wall and concentration of secretory immunoglobulin A in the intestinal mucosa. Understanding these interactions is essential for optimising the use of thymol as a natural additive to improve a healthy gut environment.

## 2. Materials and Methods

### 2.1. Experimental Design and Animal Care

After weaning at 35 days of age, a total of 32 rabbits of both sexes (an equal male-to-female ratio per treatment, meat line M91) were randomly assigned to two dietary groups: a control group (CG) receiving a standard commercial diet and an experimental group (EG) fed the same diet supplemented with thymol at a concentration of 250 mg/kg feed, with eight replicates in each (one replicate consisting of one cage with two animals). Animals were housed in wire-net cages (61 × 34 × 33 cm) under controlled environmental conditions, with a constant temperature of 22 ± 4 °C, humidity of 70 ± 5%, and a 16 h light/8 h dark photoperiod. Feed and water were provided ad libitum throughout the 28-day experimental period. At 63 days of age, eight animals per group (one animal/one replicate) were euthanised by electronarcosis (50 Hz, 0.3 A/rabbit for 5 s), immediately hung by the hind legs on the processing line and quickly bled by cutting the jugular veins and the carotid arteries.

### 2.2. Experimental Diet

The standard diet used in this study was a commercial pelleted feed for growing rabbits (KKZK, Liaharensky podnik Nitra a.s., Nitra, Slovakia), formulated to meet their nutritional requirements. The composition and chemical characteristics of the basal diet are shown in Table 1. The experimental diet was prepared by mixing the basal diet with thymol powder (≥99.9%, Sigma-Aldrich, St. Louis, MO, USA) at a concentration of 250 mg/kg feed. Both the standard and experimental diets were administered in pelleted form, with an average pellet size of 3.5 mm. To minimise oxidative degradation, the feed was stored in the dark throughout the experimental period. The diets were analysed for crude protein, ash, acid detergent fibre, starch and dry matter (DM) using standard methods of the Association of Official Analytical Chemists [12]. Thymol stability in feed was analysed weekly by high-performance liquid chromatography (HPLC) according to the modified methods [13,14]. Samples were analysed in triplicate. Briefly, 2 mL of methanol was added to a glass tube containing 0.2 g of milled feed, and thymol was extracted in an ultrasonic bath. The methanolic extract was then analysed by HPLC using an Ultimate 3000 system (Dionex, Sunnyvale, CA, USA). The chromatographic analyses were evaluated using Chromeleon^®^ Software Version 6.80 SR10 Build 2906 (Thermo Fisher Scientific, Waltham, MA, USA). The obtained thymol concentrations were relatively stable (231.0 ± 5.7–0 d, 204.2 ± 5.6–7 d, 185.0 ± 7.8–14 d, 168.8 ± 5.7–21 d µg/g DM).

### 2.3. Ethical Statement

All procedures involving animals were approved by the Institutional Ethics Committee and the State Veterinary and Food Administration of the Slovak Republic under protocol number SK U03021 (valid from 1 June 2021 until 1 June 2026). Animals were handled by qualified personnel, and all efforts were made to minimise suffering and distress before stunning, ensuring that all procedures were conducted humanely and ethically. The authors confirm that they have followed EU standards for the protection of animals used for scientific purposes.

### 2.4. Health Status and Sampling

Body weight (BW) and feed intake (FI) were monitored individually weekly throughout the trial. At the end of the experiment, average daily FI, average daily gain (ADG), and feed conversion ratio (FCR) were calculated.

For thymol and immunoglobulin A analyses, samples from the duodenum, jejunum, ileum, caecum, and proximal colon were collected, gently flushed with 0.9% saline solution, immediately frozen in liquid nitrogen, and stored at −80 °C until analysis. Approximately 5 cm of jejunum was fixed in 4% neutral buffered formalin for morphometric examination. To evaluate the intestinal microbiota, samples (approximately 1 g) of the duodenum, jejunum, ileum and caecum were collected and appropriately stored for further microbial analysis. Dry matter of individual parts of the intestinal wall was acquired by drying samples at 135 °C to a constant weight [12].

### 2.5. Analysis of Thymol in Different Parts of the Intestinal Tract

Thymol was detected in individual intestinal segments (approximately 1 g) using headspace solid-phase microextraction (HS-SPME) followed by gas chromatography coupled with mass spectrometry (GC/MS). Thymol was identified using total ion current (TIC) chromatograms and quantified by selected ion monitoring (SIM) using an Agilent 7890A gas chromatograph coupled to a 5975C VL-MSD mass selective detector with a triple-axis detector (Agilent Technologies GmbH, Waldbronn, Germany) and a CombiPAL autosampler (CTC Analytics, Agilent Technologies GmbH). The identity of thymol and the internal standard, *o*-cresol, was confirmed by comparing their retention times and mass spectra with those of reference standards. Additionally, the Kovats index was calculated. The ions monitored for quantification in SIM mode were *m*/*z* 135 and 150 for thymol and *m/z* 107 and 108 for *o*-cresol. Calibration curves were prepared using blank tissue samples (control group) spiked with known concentrations of thymol (2000 ng/g, 1000 ng/g, 800 ng/g, 400 ng/g, 200 ng/g, 100 ng/g). For each intestinal section, the calibration curves were constructed by plotting the peak area ratio of thymol to the internal standard against the known thymol concentrations. Each calibration point was measured as a duplicate. *β*-Glucuronidase from *Helix pomatia* Type HP-2 (aqueous solution, ≥100,000 units/mL; Sigma-Aldrich, St. Lo, France) was added to the samples to cleave thymol from its glucuronide and sulphate conjugates, as only free thymol could be detected by the GC system [15].

### 2.6. Jejunal Morphometry Investigation

The evaluated morphometric indices were villus height (Vh—villi tip to base of the villi), crypt depth (Cd—base of the villi to bottom of the crypt) and the ratio of villi height to crypt depth (Vh/Cd) [16]. The samples (approximately 3–4 cm) were processed according to a modified procedure based on previously published methods [17,18]. Briefly, samples were embedded in paraffin, sectioned into 10 serial slices (5 μm thick), stained with haematoxylin and eosin, and examined by light microscopy.

### 2.7. Microbial Evaluation in Different Parts of the Intestinal Tract

Samples (approximately 1 g) from the duodenum, jejunum, ileum, and caecum were processed using the standard microbiological dilution technique in accordance with the guidelines of the International Organisation for Standardisation (ISO). Serial dilutions in Ringer’s solution (pH 7.0; Oxoid Ltd., Hampshire, UK) were prepared and cultivated as described by Pogány Simonová et al. [19]. Cultivation was carried out at 30 °C and/or 37 °C for 24–48 h, depending on the bacterial genus. Microbial counts were expressed as the log10 of colony-forming units per gram of sample (log10 CFU/g ± SD). Randomly selected colonies representing specific bacterial groups were identified using the MALDI-TOF identification system (Bruker Daltonics, Bremen, Germany).

### 2.8. Immunoglobulin A Investigation in Different Parts of the Intestinal Tract

Intestinal wall samples (approximately 50 mg/one sample) were prepared as described by Nikawa et al. [20]. Quantitative determination of rabbit IgA in the intestinal wall was performed using a competitive inhibition enzyme-linked immunosorbent assay (Cusabio ELISA kit, Cat. No. CSB-E06946Rb, CUSABIO Biotech Co., Ltd., Wuhan, China). Standards and samples were added to a 96-well microplate precoated with a horseradish peroxidase-conjugated antibody specific for rabbit IgA.

### 2.9. Statistical Analysis

Differences in thymol concentrations between the duodenum, jejunum, ileum and colon were analysed using analysis of variance (ANOVA). The prerequisites for ANOVA, particularly the normal distribution of the residuals, were checked using QQ plots and the Shapiro–Wilk normality test. The homogeneity of the variances was evaluated using the Levene test. Statistical analysis was performed in R-Studio version 2023.3.0.386. (Posit Software, Boston, MA, USA, http://www.posit.co/). The data collected for morphometric parameters, IgA concentrations, and microbial counts were analysed using the Shapiro–Wilk normality test. The differences between the control and experimental groups for morphometric parameters in the jejunum; microbial counts in the duodenum, jejunum, ileum, and caecum; and immunoglobulin A concentration in the duodenum, jejunum, ileum, caecum and colon were evaluated using the Mann–Whitney U test. Results are presented as mean ± standard deviation (SD). Differences were considered statistically significant at *p* < 0.05. Statistical analyses were performed using GraphPad Prism version 10.4.2 for Windows (GraphPad Software, Boston, MA, USA, http://www.graphpad.com/).

## 3. Results

Not all data were accepted as parametric.

### 3.1. Growth Performance

The animals remained in good health throughout the experimental period, and no differences in performance were observed between the CG and EG groups. The final slaughter weight was 2646 ± 131.6 g (CG) and 2653 ± 116.2 g (EG), ADG 37.7 ± 6.5 g/day/rabbit (CG) and 38.7 ± 2.5 g/day/rabbit (EG), FCR 3.5 ± 0.4 (CG) and 3.5 ± 0.3 (EG).

### 3.2. Thymol in Different Parts of the Intestinal Tract

**Figure 1:**
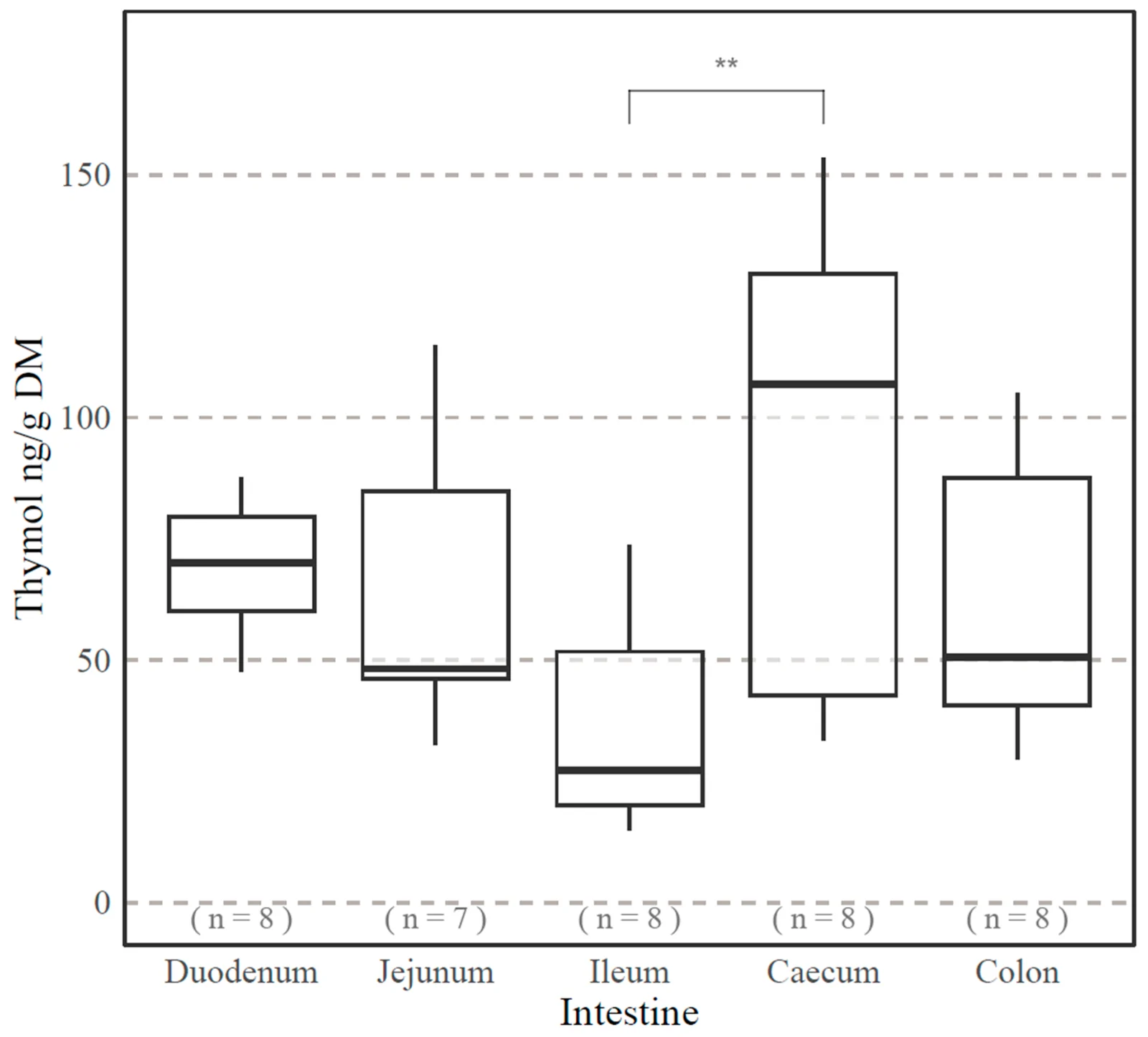
Thymol in different parts of the rabbit intestinal tract (n = 8). Footnotes: One-way analysis of variance (ANOVA). Data are presented as Medians with interquartile ranges (boxes) and minimum-maximum values (whiskers). Double asterisk (**) represents a significance level of *p* < 0.01. Abbreviation: DM, dry matter.

Thymol concentration was significantly higher in the caecum than in the ileum (*p* = 0.038) and was balanced in the duodenum, jejunum and proximal colon (Figure 1).

### 3.3. Jejunal Wall Morphometry

Morphometric indices of the jejunal wall were not significantly affected by thymol treatment (Table 2).

### 3.4. Microbial Evaluation in Different Parts of the Intestinal Tract

**Figure 2:**
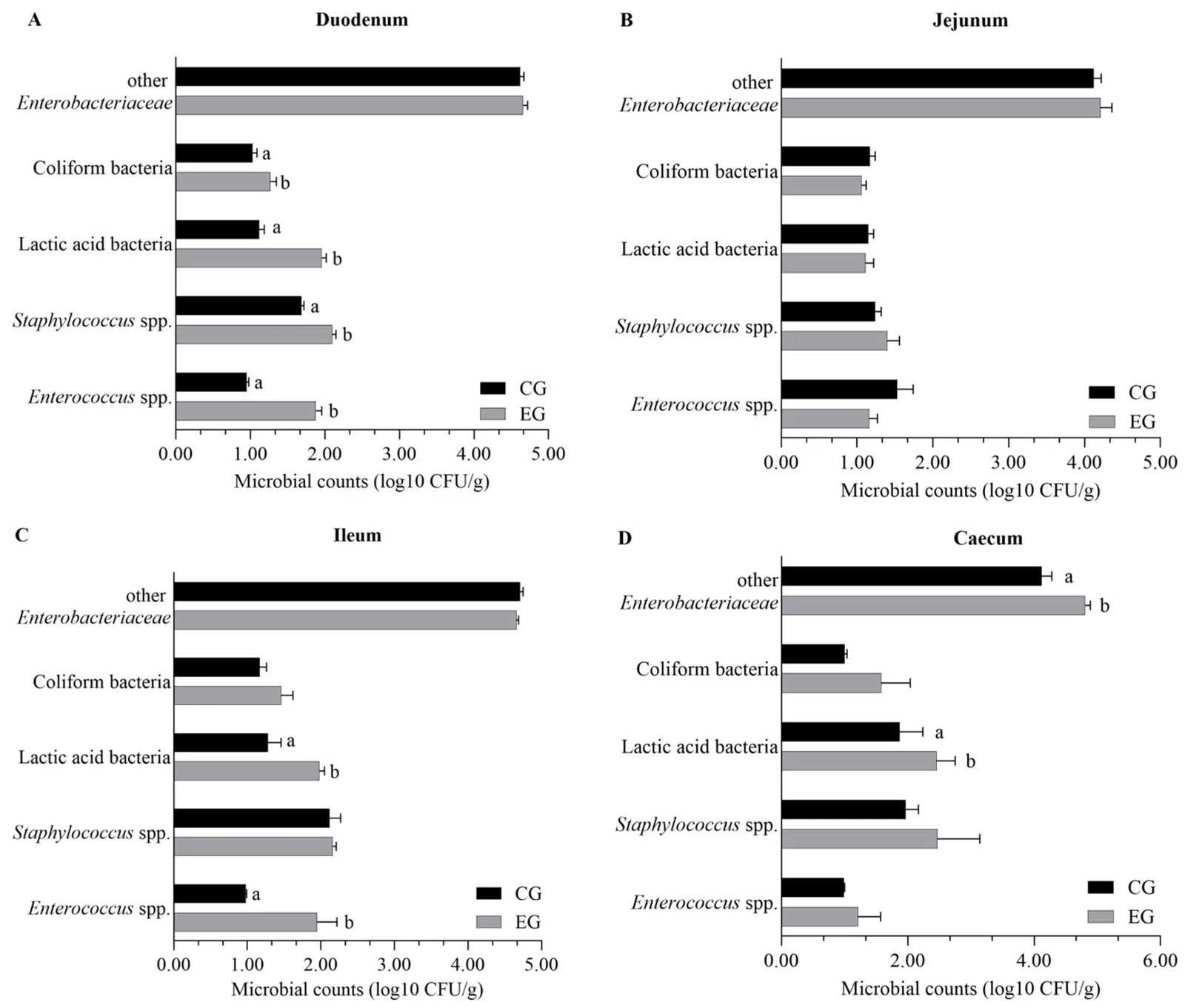
(**A**–**D**) Effect of thymol on microbial counts in different intestinal parts of rabbits (n = 8). Footnotes: Data are presented as mean ± standard deviation (SD). Different superscript letters (^a, b^) differ significantly at *p* < 0.05. Abbreviation: spp., species; CG, control group; EG, experimental group.

Coliform bacteria (*p* = 0.0095) and lactic acid bacteria (LAB; *p* = 0.0002), *Staphylococcus* spp. (*p* = 0.0002), and *Enterococcus* spp. (*p* = 0.0002) in the duodenum (Figure 2A); LAB (*p* = 0.0003) and *Enterococcus* spp. (*p* = 0.0002) in the ileum (Figure 2C); and other *Enterobacteriaceae* (*p* = 0.0002) and LAB (*p* = 0.0068) in the caecum were significantly higher in the EG (Figure 2D). In the jejunum, no bacterial genera examined exhibited statistically significant differences between the groups (Figure 2B).

### 3.5. Immunoglobulin A in Different Parts of the Intestinal Tract

**Figure 3:**
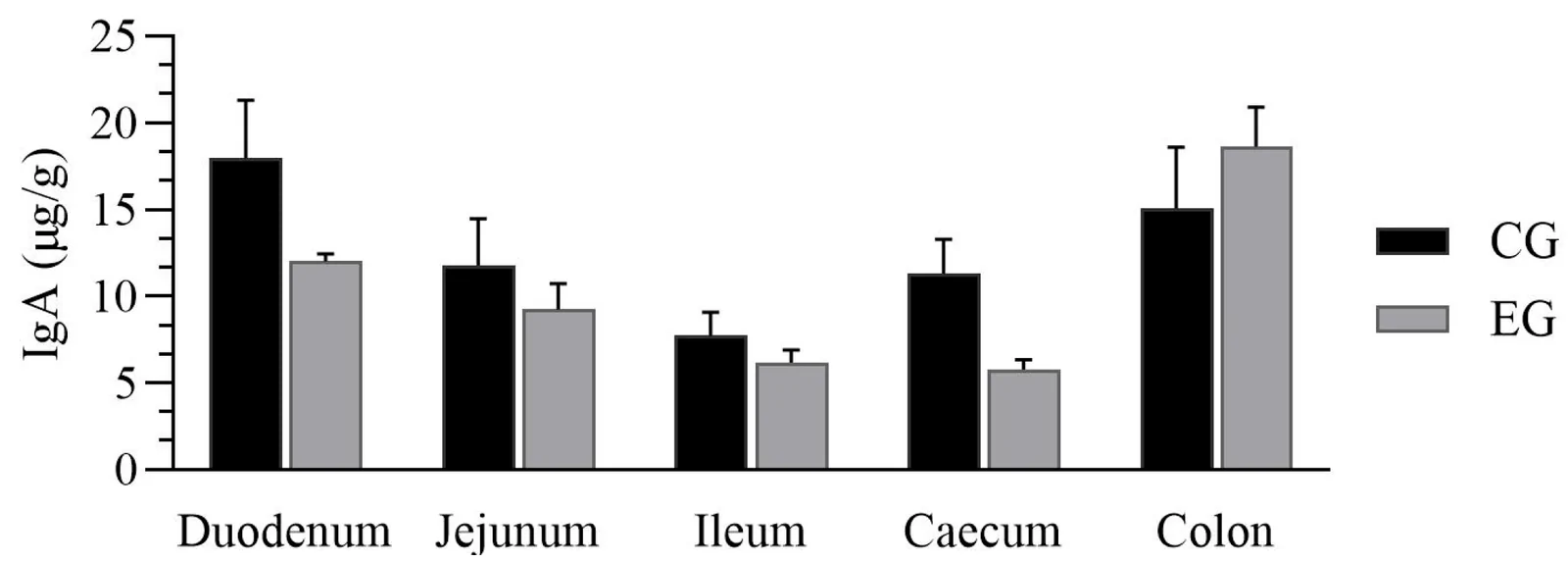
Effect of thymol on IgA concentration in different intestinal parts of rabbits (n = 8). Footnotes: Data are presented as mean ± standard deviation (SD). Abbreviation: IgA, immunoglobulin A; CG, control group; EG, experimental group.

No statistically significant differences in IgA concentrations were observed among EG across gastrointestinal segments. However, a decreasing trend was observed in the EG compared to the CG in all intestinal segments except the colon (*p* > 0.05; Figure 3).

## 4. Discussion

The distribution of thymol throughout the gastrointestinal tract is likely influenced by several physiological processes and biological barriers affecting its absorption, metabolism, and excretion. One of the most important factors affecting the fate of orally administered compounds is first-pass metabolism, which occurs in both the intestinal wall and the liver. Following absorption from the intestinal lumen, compounds undergo phase I and phase II biotransformation within enterocytes. The unmetabolized fraction is either transported back into the intestinal lumen or transferred via the mesenteric veins into the circulation. The parent compound or its metabolites are subsequently transported to the liver, where they undergo further metabolism and may be excreted into bile and returned to the duodenum. This process contributes to the presystemic elimination of orally administered compounds, thereby influencing the overall bioavailability of thymol [21].

The relatively comparable concentrations of thymol observed in the duodenum, jejunum, and colon in our study can be explained by the metabolic processes described above, as well as by the continuous delivery of thymol and its metabolites to the duodenum via bile. According to Varga and Harcourt-Brown [22], the bile duct in rabbits opens directly into the proximal duodenum, which may contribute to the relatively higher concentrations of thymol observed in this segment compared to the jejunum and colon, where luminal levels appear more equally distributed. As described by Bacova et al. [11], thymol and its metabolites are rapidly absorbed in rabbits across the intestinal epithelium into the portal circulation and transported to the liver, where they undergo biotransformation processes and are subsequently excreted into the bile, returning to the duodenum. This process allows for potential reabsorption of thymol compounds and represents a classic example of enterohepatic recirculation. Moreover, in the lower part of the small intestine, especially the ileum, conjugated drugs and bile salts are reabsorbed into the hepatic portal circulation. Bacterial enzymes in the small intestine can deconjugate certain compounds, thereby converting them back into their lipid-soluble parent forms and facilitating their reabsorption [23]. Dieterich, Schink, and Zopf [24] reported that the variability and abundance of the intestinal microbiota increase progressively from the stomach through the duodenum, jejunum, and ileum to the colon. The slightly lower thymol concentration observed in the ileum may be attributed to the enzymatic activity of the intestinal microbiota. This finding may be explained by thymol’s increased tendency to interact with the gut microbiota and subsequently undergo enterohepatic circulation, as suggested by Sousa et al. [25]. Furthermore, Skalova et al. [26] and Nakanishi et al. [27] reported that metabolic enzyme activity within the intestinal epithelium decreases from proximal to distal intestinal segments. This observation is consistent with our findings and may explain the lower thymol concentration detected in the ileum, potentially due to reduced conjugation activity and, consequently, greater absorption of lipophilic thymol across the intestinal wall into the systemic circulation (Figure 1).

The cecum represents the largest compartment of the rabbit’s large intestine and is the primary site of microbial fermentation. Due to cecotrophy, in which undigested material is recycled from the proximal colon back to the cecum, a continuous supply of substrates is provided to the caecal microbiota, supporting intensive fermentative activity. These processes may influence thymol concentrations within this intestinal segment. Bacova et al. [11] attributed the high thymol concentrations detected in the cecum to extensive enzymatic activity associated with the highly diverse caecal microbiota, leading to intensive biotransformation of thymol in both caecal contents and cecotrophs. The authors further suggested that compounds and metabolites that cannot cross the intestinal epithelium in the small intestine, where absorption is most intensive, are transported to more distal regions of the gastrointestinal tract and eventually excreted in faeces. The elevated thymol concentrations observed in the cecum in the present study are consistent with these findings. Nevertheless, the biotransformation of thymol within the intestinal wall, as well as the involvement of intestinal enzymes, transporters, and receptors, has not yet been fully elucidated.

Thymol is a lipophilic compound known for its strong antibacterial activity against both Gram-positive and Gram-negative bacteria, such as *Staphylococcus aureus* [28], *Pseudomonas aeruginosa* [29], *Escherichia coli* and *Listeria monocytogenes* [30] and *Aeromonas hydrophila* [31]. Its primary mode of action involves disrupting bacterial cell membranes, where it integrates into the lipid bilayer and accumulates within fatty acid chains. This leads to conformational changes, loss of membrane integrity and increased permeability, ultimately causing leakage of intracellular components and bacterial cell death [32]. While Gram-negative bacteria are generally more resistant due to a rigid outer membrane rich in lipopolysaccharides, which limits the penetration of hydrophobic compounds, Gram-positive bacteria lack this barrier. Instead, they possess a thick but porous peptidoglycan layer and lipoteichoic acids with lipophilic ends that may facilitate the diffusion of thymol toward the cell membrane [33]. Although increased membrane permeability is considered the main mechanism of thymol action, its ability to cross membranes also suggests potential intracellular targets. The antibacterial effectiveness of thymol, therefore, depends not only on its physicochemical properties but also on the structural characteristics of the bacterial cell wall and the surrounding environment [34].

Mahran et al. [35] highlighted the strong antioxidant properties of thymol, which are attributed to its phenolic structure, with the hydroxyl group (–OH) playing a key role in free radical scavenging. In our experiment, thymol supplementation tended to increase the counts of the examined bacterial populations throughout the intestinal tract (Figure 2A–D). Based on these findings, it can be assumed that the concentration of thymol in the intestinal lumen did not induce oxidative damage to the cell walls of the examined bacteria but instead exhibited antioxidant activity. The significant increase in LAB observed in our study suggests enhanced production of short-chain fatty acids, which subsequently reduced the pH of the intestinal environment. Lactobacilli are strong producers of lactic acid and therefore represent effective competition for pathogenic bacteria, as an acidic environment inhibits their growth [36]. Cremonesi et al. [37] reported that supplementation with Goji berries stimulated fermentation, thereby altering the composition of the gut microbiota toward a more beneficial bacterial profile. Similarly to these observations, our results demonstrated a positive effect of thymol supplementation on the composition of the gut microbiota.

The mucosal immune system serves as the first line of defence against infections entering through mucosal surfaces, where a delicate balance among immune cells, secretory molecules and the gut microbiota is essential for maintaining gut health. As the dominant antibody isotype at mucosal surfaces, Secretory IgA (sIgA) plays a central role in this process by limiting the adherence of commensal microorganisms to the intestinal epithelium while preventing the invasion of pathogenic microorganisms across the epithelial barrier. The gut microbiome comprises a diverse community of beneficial commensals and potential pathogens, requiring the immune system to carefully distinguish between these populations. Through its interactions with the intestinal microbiota, sIgA promotes tolerance toward commensal microorganisms while supporting protective immune responses against pathogens, thereby contributing to mucosal homeostasis and resistance to infection [38].

Du et al. [39] demonstrated that broilers challenged with *C. perfringens* exhibited a significant increase in mucosal sIgA levels (*p* < 0.05), reflecting the stimulation of mucosal immune responses to pathogen invasion. However, supplementation with essential oils containing 25% thymol and 25% carvacrol did not significantly affect sIgA secretion. This finding suggests that these essential oils may have supported intestinal barrier integrity rather than inducing an enhanced mucosal immune response, thereby reducing antigen exposure and subsequent inflammatory signalling. Similarly, in our study, thymol did not increase sIgA production but instead tended to reduce sIgA secretion across various intestinal segments except in the colon in rabbits (Figure 3). Lower sIgA concentrations do not necessarily indicate an adverse effect on intestinal health. Increased sIgA secretion is often associated with greater antigenic stimulation and activation of mucosal immune responses. Therefore, the reduced sIgA levels observed in the present study may reflect lower stimulation of the mucosal immune system and improved intestinal homeostasis [40]. Moreover, the increased LAB counts observed in the experimental group may have contributed to a more balanced intestinal environment, thereby reducing the need for an enhanced mucosal immune response. However, no decreasing trend was observed in the colon, suggesting that local microbial and immunological conditions in this intestinal segment may differ from those in the small intestine. This finding indicates that the effect of thymol on mucosal immunity may vary along the intestinal tract. These findings support a beneficial role of thymol in maintaining intestinal homeostasis by promoting intestinal barrier integrity, preserving microbial balance, and modulating mucosal immune responses.

## 5. Conclusions

Among the main findings of our experimental study was that thymol concentration was lowest in the ileum, likely due to increased enzymatic activity of the intestinal microbiota, followed by enterohepatic circulation. In contrast, the highest concentration was detected in the caecum due to caecotrophy, which promoted thymol redistribution and enhanced its bioavailability in the rabbits. However, thymol concentration in the intestinal tract did not significantly affect sIgA secretion; instead, it tended to reduce its production. Nevertheless, its interactions with the intestinal microbiota support protective immune responses against pathogens. Thymol also positively influenced the composition of the gut microbiota by increasing LAB counts throughout the intestinal tract, thereby creating an unfavourable environment for the adhesion of pathogenic bacteria to the intestinal wall. The concentrations of thymol throughout the intestinal tract helped maintain intestinal homeostasis, thereby supporting the functional integrity of the intestinal barrier and modulating the mucosal immune response. Our findings highlight the importance of gaining a deeper understanding of these interactions to optimise thymol concentrations and thereby support the maintenance of a healthy intestinal environment.

## Figures and Tables

**Table 1:** Ingredients and chemical composition (g/100 g DM) of experimental diets (dry matter—DM basis).

Ingredients	g/100 g DM	Composition	g/100 g DM
Dehydrated lucerne meal	33.7	Dry matter (g/100 g)	93.7
Dry malting sprouts	14.1	Organic compounds	77.9
Oats	12.2	Nitrogen-free extract	41.6
Wheat bran	8.4	Neutral detergent fibre	33.1
Barley	7.5	Acid detergent fibre	19.5
Extracted sunflower meal	5.2	Crude fibre	16.7
Extracted rapeseed meal	5.1	Crude protein	16.6
Dried distiller’s grains with solubles	4.7	Cellulose	15.3
Premix ^1^	1.6	Hemicellulose	13.6
Limestone	0.9	Starch	12.5
Sodium chloride	0.3	Ash	6.5
Total	93.7	Fat	3.1
		Metabolic energy, MJ/kg	9.91

^1^ The vitamin-mineral premix provided per 100 g of complete diet: Retinyl acetate 0.52 mg, Cholecalciferol 0.003 mg, Tocopherol 0.003 mg, Thiamine 0.08 mg, Riboflavin 0.30 mg, Pyridoxin 0.20 mg, Cyanocobalamin 0.002 mg, Niacin 0.38 mg, Folic acid 0.06 mg, Calcium 0.18 mg, Iron 0.70 mg, Zinc 0.66 mg, Copper 0.15 mg, Selenium 0.03 mg. Abbreviation: DM, dry matter.

**Table 2:** Effect of thymol on morphometric indices in the jejunal wall of rabbits (n = 8).

Parameter	CG	EG
Vh (µm)	688.90 ± 12.45	696.50 ± 10.86
Cd (µm)	179.00 ± 6.23	175.80 ± 5.70
Vh/Cd	3.86 ± 0.15	3.97 ± 0.16

Footnote: Data are presented as mean ± standard deviation (SD). Abbreviations: Vh, villi height; Cd, crypt depth; Vh/Cd, villi height to crypt depth ratio; CG, control group; EG, experimental group.

## Data Availability

The data that support the findings of this study are available upon request from the corresponding author.
